# A Reliable Remedy for Ozæna

**Published:** 1886-03

**Authors:** 


### A Reliable Remedy for Ozæna.

Besides the removal of the diseased bone, the only remedy which has thus far been considered to possess any value in ozæna has been chloride of zinc, and every physician will admit that its effect never answers the expectation.

A new preparation of aluminium, the aluminium acetico-tartaricum, has been discovered to be almost a specific in ozæna. Altenstart and Schaefer (*Deutsche Med. Woch.*, 23, 85,) have tried it in a large number of cases, and say that its effect is really surprising. The fœtor of the secretion rapidly disappears, the scabs become thinner under its use and drop off much easier, the atrophic mucous membrane assumes a healthy look, and within two weeks a complete cure may be established in all cases where the bone has not already been so diseased as to be loose like a sequestrum. Of a fifty per cent. solution, one teaspoonful is usually added to one-half to one pint of water. It possesses about the same caustic properties as a solution of nitrate of silver of one to five, and is also indicated in the same morbid conditions of the mucous membrane as the latter drug, but it seems to have a specific effect upon the mucous membrane of the nose and of the larynx. In ulcerations, for instance, of the larynx, its effect is far more rapid than that of boric acid.

We do not know whether the remedy has reached this country, but it will be rapidly imported whenever some of our leading physicians conclude to make a trial of it.

